# Usefulness of bicarbonate-based Impella purge solution in a patient with heparin-induced thrombocytopenia: the first case report of long-term management in Japan

**DOI:** 10.1007/s10047-024-01452-0

**Published:** 2024-06-06

**Authors:** Shin Nagai, Hiroaki Hiraiwa, Ryota Ito, Yuichiro Koyama, Kiyota Kondo, Shingo Kazama, Toru Kondo, Ryota Morimoto, Takahiro Okumura, Hideki Ito, Tomo Yoshizumi, Masato Mutsuga, Toyoaki Murohara

**Affiliations:** 1 Department of Cardiology, Nagoya University Graduate School of Medicine, 65 Tsurumai-cho, Showa-ku, Nagoya, 466-8550 Japan; 2 Department of Cardiac Surgery, Nagoya University Graduate School of Medicine, 65 Tsurumai-cho, Showa-ku, Nagoya, 466-8550 Japan

**Keywords:** Impella, Bicarbonate-based purge solution, Heparin-induced thrombocytopenia, Thrombosis, Heart failure, Off-label use

## Abstract

Percutaneous mechanical circulatory support utilizing micro-axial flow pumps, such as the Impella group of devices, has become a life-saving technique in the treatment of refractory cardiogenic shock, with ever-increasing success rates. A 30-year-old man presented with acute decompensated heart failure and a severely reduced left ventricular ejection fraction (17%). Despite initial treatment with inotropic drugs and intra-aortic balloon pump support, his hemodynamic status remained unstable. Transition to Impella CP mechanical circulatory support was made on day 6 owing to persistently low systolic blood pressure. A significant decline in platelet count prompted suspicion of heparin-induced thrombocytopenia (HIT), later confirmed by positive platelet-activated anti-platelet factor 4/heparin antibody and a 4Ts score of 6 points. Argatroban was initially used as the purge solution, but owing to complications, a switch to Impella 5.0 and a bicarbonate-based purge solution (BBPS) was performed. Despite additional veno-arterial extracorporeal membrane oxygenation support on day 24, the patient, aiming for ventricular assist device treatment and heart transplantation, died from infection and multiple organ failure. Remarkably, the Impella CP continued functioning normally until the patient’s demise, indicating stable Impella pump performance using BBPS. This case highlights the usefulness of BBPS as an alternative to conventional Impella heparin purge solution when HIT occurs.

## Introduction

The Impella ventricular support system (Abiomed, Inc, Danvers, MA), which is a percutaneous ventricular assist device (VAD), is an invaluable resource in handling refractory cardiogenic shock, and its use has notably surged in the past decade [1]. One unique factor of Impella devices is the continuous flow release of a dextrose-based purge solution containing unfractionated heparin (UFH) from the motor housing to prevent device-related clotting. However, owing to contraindications in some patients (for instance heparin-induced thrombocytopenia [HIT]) or cases of severe bleeding, the manufacturer has recently secured Food and Drug Administration (FDA) approval to use a bicarbonate-based purge solution (BBPS) as an alternative to UFH in the purge. This report highlights a case of a 30-year-old male patient whose long-term management with Impella CP support was achieved using BBPS following the development of HIT.

## Case presentation

A 30-year-old man was admitted to a nearby hospital for acute decompensated heart failure. On admission to that hospital, echocardiography showed that the left ventricular end-diastolic diameter (LVDd) was 62 mm and left ventricular ejection fraction (LVEF) was only 17%. The patient had left and right ventricular enlargement, moderate mitral regurgitation (MR), and moderate tricuspid regurgitation (TR). Tricuspid annular plane systolic excursion (TAPSE), a measure of right heart systolic function, was reduced to 10 mm. He was diagnosed with dilated cardiomyopathy based on normal coronary angiography findings and myocardial pathology examination of a right ventricular myocardial biopsy. The patient experienced cardiogenic shock, which was caused by acute decompensated heart failure associated with dilated cardiomyopathy. This was his first admission for a heart condition. Inotropic drugs (dobutamine 4.0 µg/kg/min, milrinone 0.25 µg/kg/min) were administered, but his symptoms did not improve. Thus, intra-aortic balloon pump (IABP) was added for more support and the patient was transferred to our hospital (day 1).

**Fig. 1:**
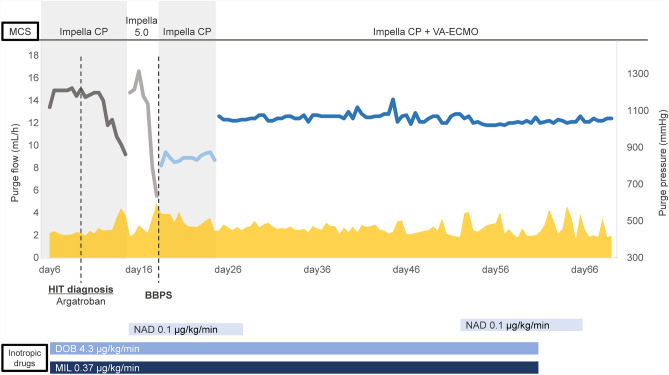
Trends in Impella purge flow and pressure. BBPS, bicarbonate-based purge solution; DOB, dobutamine; HIT, heparin-induced thrombocytopenia; MCS, mechanical circulatory support; MIL, milrinone; NAD, noradrenaline; VA-ECMO, veno-arterial extracorporeal membrane oxygenation

**Fig. 2:**
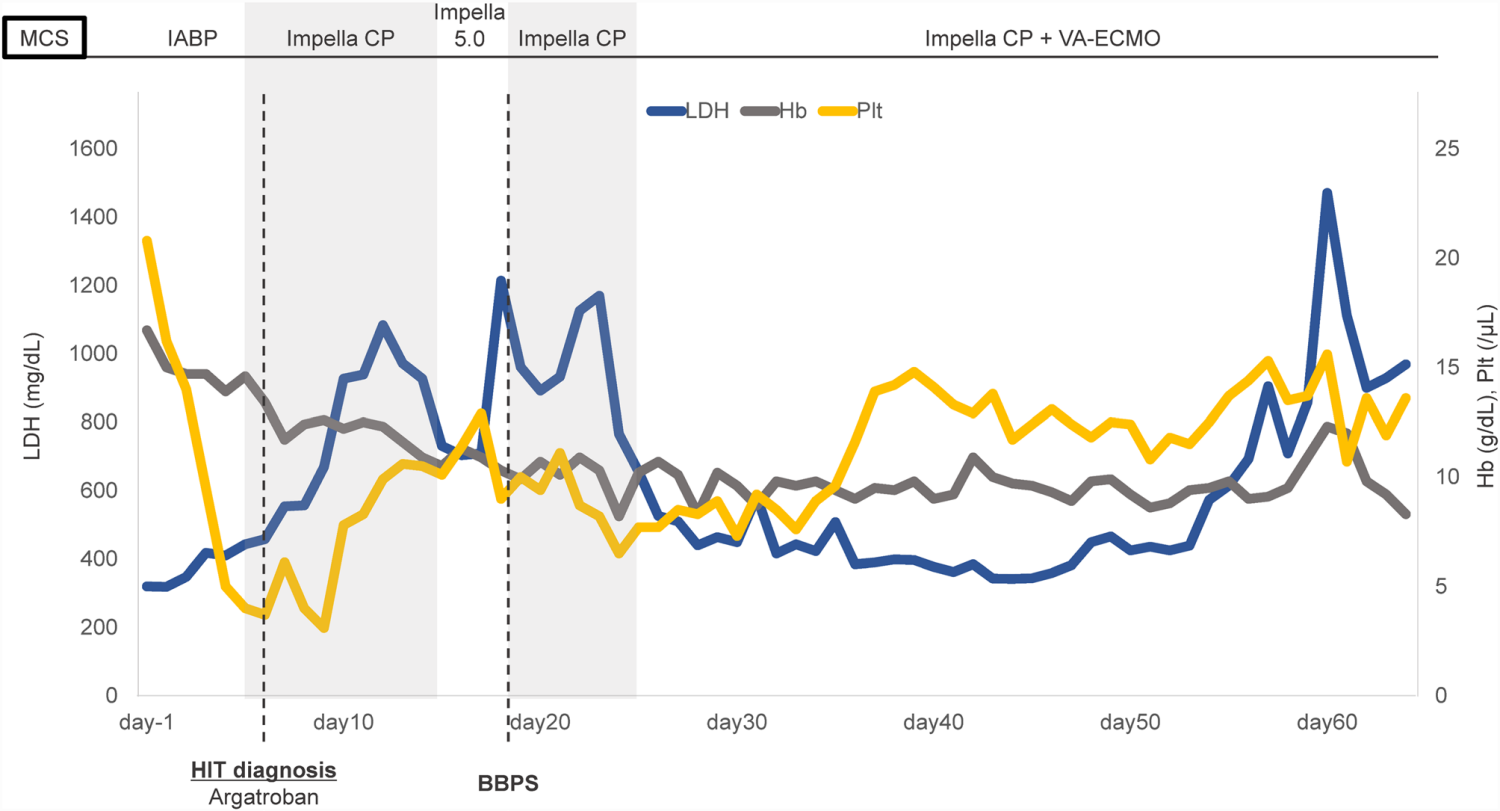
Trends in blood lactate dehydrogenase, hemoglobin, and platelet count. BBPS, bicarbonate-based purge solution; Hb, hemoglobin; HIT, heparin-induced thrombocytopenia; LDH, lactate dehydrogenase; MCS, mechanical circulatory support; Plt, platelet; VA-ECMO, veno-arterial extracorporeal membrane oxygenation

After transfer to our hospital, the patient’s height, weight, and body surface area were 165 cm, 72 kg, and 1.79 m^2^, respectively, and echocardiography showed an LVDd of 64 mm and an LVEF of 23% on day 1. Even with IABP support, his systolic blood pressure was still below 80 mmHg and he was not hemodynamically stable, with hepatic dysfunction (aspartate aminotransferase, 37 U/L; alanine aminotransferase, 90 U/L; γ-glutamyltranspeptidase, 73 U/L; total bilirubin, 3.7 mg/dL) and renal dysfunction (blood urea nitrogen, 22.0 mg/dL; creatinine, 1.11 mg/dL; estimated glomerular filtration rate, 65.2 mL/min/m^2^) observed on day 6. Owing to worsening of the patient’s hemodynamic status by infection at the catheter site on day 6, we upgraded the mechanical circulatory support (MCS) from IABP to Impella CP (Fig. 1), which was placed via the left femoral artery approach on day 6. Given his relatively small stature, we determined that the Impella CP would be sufficient for MCS. After replacing the IABP with the first Impella CP, LVDd was 60 mm and LVEF was 12% on day 6. We used the recommended purge solution composition (heparin 25 U/mL, 5% dextrose solution), and the purge flow rate was approximately 14.9 mL/h. Meanwhile, the platelet count decreased steadily from 162,000/L at the time of transfer to 31,000/L on day 9, and laboratory data revealed platelet-activated anti-platelet factor 4/heparin antibody > 5.0 U/mL (normal range < 1.0 U/mL) (Fig. 2). Heparin had been used before the patient was transferred to our hospital, and after his transfer, heparin was continued. Therefore, this event occurred on day 15 of heparin use. The 4Ts Score of 6 points (Thrombocytopenia, 2 points; Timing of platelet count fall, 2 points; Thrombosis of other sequelae, 1 point; Other causes of thrombocytopenia, 1 point) indicated a high possibility of HIT (6 points). Finally, the diagnosis of HIT was confirmed by a hematologist. As systemic anticoagulation therapy, heparin (10,000–15,000 U/day) was used until HIT was identified. After the discovery of HIT, argatroban (0.3–0.35 µg/kg/min) was used for systemic anticoagulation.

After changing to argatroban as the alternative purge solution (argatroban 0.05 mg/mL, 5% dextrose solution) on day 14, the purge flow showed an abrupt decline to 9.2 mL/h, and a high risk of emergency shutdown of the Impella CP pump was indicated. Therefore, we changed the device to Impella 5.0 (Fig. 1), which was placed via the right femoral artery approach on day 14. We choose Impella 5.0 instead of Impella 5.5 because the subclavian artery diameter was only 6.5 mm on blood vessel echocardiography and computed tomography, which would have made the Impella 5.5 approach challenging. After switching to Impella 5.0, LVDd was 59 mm and LVEF was 18% on day 14. However, the purge flow of Impella 5.0 again showed a decline to 5.5 mL/h on day 18, likely owing to Impella pump thrombus, so we switched the device to the second Impella CP, which was placed via the left femoral artery approach on day 18. We also changed the purge solution from an argatroban solution to the BBPS (25 mEq/L sodium bicarbonate, 5% dextrose solution). After switching Impella 5.0 to the second Impella CP, LVDd was 66 mm, LVEF was 21%, and TAPSE was 12.6 mm on day 18. After switching to BBPS, the purge flow of Impella CP could be sustained for more than 7 weeks. The purge flow and pressure were approximately 9–12 mL/h and 400–500 mmHg, respectively. As well as MCS by Impella CP (2.5 L/min), inotropic drugs were used. However, the cardiac index was 1.6 L/min/m^2^, mixed venous oxygen saturation was 59%, and mean blood pressure was 70 mmHg. Moreover, there was progression to multiorgan damage with hepatic dysfunction, renal dysfunction, and decreased urine output (20–30 mL/h), and the patient was determined to be in a low cardiac output state.

Therefore, veno-arterial extracorporeal membrane oxygenation (VA-ECMO) was added as MCS via the left femoral arteriovenous approach, along with the third Impella CP (Fig. 1) placed via the right subclavian artery approach on day 24 to maintain systemic hemodynamics. After the addition of VA-ECMO support, the patient’s circulatory status stabilized and was maintained at an approximate Impella CP pump flow of 2.1 L/min and VA-ECMO flow of 3.3 L/min. As a result, the cardiac index increased to 2.0 L/min/m^2^, mixed venous oxygen saturation increased to 80%, and mean blood pressure increased to 78 mmHg. Organ damage improved and urine output increased (60–80 mL/h). On day 24, LVDd was 60 mm, LVEF was 23%, and TAPSE was 15.2 mm. Thereafter, LVDd, LVEF, right ventricular contraction, and degree of MR and TR were maintained without deterioration under stable MCS with the use of BBPS. Despite the addition of VA-ECMO to Impella CP as MCS, there was no exacerbation of hemolysis, and the patient could be managed for approximately 1 month (Fig. 2). There were no adverse events associated with BBPS, such as alkalosis, hypernatremia, or other electrolyte abnormalities.

Unfortunately, the patient died on day 69 owing to infection and consequent disseminated intravascular coagulation, resulting in multiple organ failure while awaiting implanted VAD treatment with the expectation of a heart transplant. Nonetheless, Impella CP was still functioning normally when the patient died, with a purge flow of 12.4 mL/h and a purge pressure of 420 mmHg.

## Discussion

There have been two prior reports on the use of BBPS for confirmed HIT [2, 3] (Table 1); however, we report the first case in Japan. The patient described here experienced cardiogenic shock complicated by severe heart failure and HIT and achieved long-term Impella-driven stabilization by transitioning from conventional UFH purge fluid to BBPS. The necessity of systemic anticoagulation when using Impella to prevent pump-related clotting poses an increased bleeding risk, exacerbated by factors such as sepsis, thrombocytopenia, and acquired von Willebrand syndrome due to elevated shear stress. Current data on anticoagulation management with Impella are limited, emphasizing the need for standardized approaches [1].

**Table 1:** Literature summary showing the characteristics and reasons for BBPS while on Impella

Author and year	Study design	No. of pts	Sex	Age (years)	BSA (m^2^)	Reason for Impella	Impella type	No. of Impella devices	No. of pts who switched from heparin to BBPS	Reason for BBPS over heparin	Duration of Impella use with BBPS	Complications while on BBPS	Outcome
Al-Ayoubi et al., 2023 [2]	Case series	2	Both male	Case 1: 48; Case 2: 69	NR	Case 1: cardiogenic shock with HFrEF, DCM, and chronic severe MR; Case 2: cardiogenic shock secondary to NSTEMI and cardiac arrest	CP	1 device each	2	HIT	Case 1: ~ 13 days (PODs 2–15 until Impella explant); Case 2: 11 days until death	None	Case 1: discharge and Impella explant; Case 2: death after family withdrawal of care
Sigala et al., 2024 [3]	Case series	18	Male n = 13 (72%)	Median 58 (IQR 48–64)	NR	Cardiogenic shock in ACS n = 5 (28%), cardiogenic shock in DHF n = 5 (28%), bridge to LVAD n = 3 (17%), bridge to OHT n = 2 (11%), postcardiotomy shock n = 3 (17%)	CP n = 9 (50%), 5.5 n = 9 (50%)	Concomitant MCS with Impella RP heart pump n = 1 pt (6%)	n = 7 (39%)	Coagulopathy n = 5 (28%) Suspected HIT n = 2 (11%) Confirmed HIT n = 1 (6%) Major bleeding n = 10 (56%)	CP: median 2.9 (IQR 1.7–5.8) days; 5.5: median 8.9 (IQR 3.0–35.9) days	n = 3 pts (17%) (pump exchange n = 1; Impella removal n = 1; alteplase for suspected purge block n = 1); of these, 2 experienced complications > 21 days into BBPS therapy	OHT n = 2 (11.1%), recovery n = 7 (38.9%), death n = 6 (33.3%), LVAD n = 3 (16.7%)
Simonsen et al., 2021 [10]	Case report	1	Female	41	NR	Infarction of the LAD coronary artery with subsequent AHF	5.5	2 devices (central Impella 5.5 followed by peripheral Impella 5.5 to enable tracheostomy)	1	Presence of thrombocytopenia with concern over HIT	7 days until Impella became dislodged	Second device (peripheral Impella 5.5) became dislodged, necessitating replacement with IABP. However, no pump thrombosis or severe bleeding events, and no hypernatremia or mild systemic alkalosis occurred	Intracorporeal VAD
Van Edom et al., 2023 [12]	Case report	1	Female	73	NR	Cardiogenic shock due to acute HFrEF	CP	1 device	1	Active hemothorax	48 h	No signs of pump thrombosis, rising purge pressures, or hemolysis	Weaned from Impella on day 19; died on day 35 due to infection
Bergen et al., 2023 [17]	Single-center, retrospective study	43	Male n = 33 (76.7%)	Median 59 (IQR 50–66)	NR	Cardiogenic shock	BBPS group: CP, n = 11 (25.6%) 5.5, n = 31 (72.1%) CP + RP, n = 1 (2.3%)	n = 2 pts (4.7%) required Impella pump replacement	NR	Indications for BBPS not specified	With BBPS: median 6.6 (IQR 4.4–11.0) days; pre-BBPS: median 0.9 (IQR 0.6–5.9) days	Thrombosis events n = 12 (27.9%), including multiple purge pressures > 800 mmHg n = 7 (16.3%), thrombolytics in purge n = 3 (7.0%), and Impella replacement n = 2 (4.7%)	NR
Present case	Case report	1	Male	30	1.79	ADHF associated with DCM	CP, 5.0	4	1	HIT	52 days	None	Death due to infection and consequent disseminated intravascular coagulation with multi-organ failure

*ACS* acute coronary syndrome, *ADHF* acute decompensated heart failure, *AHF* acute heart failure, *BBPS* bicarbonate-based purge solution, *BSA* body surface area, *DCM* dilated cardiomyopathy, *DHF* decompensated heart failure, *HFrEF* heart failure with reduced ejection fraction, *HIT* heparin-induced thrombocytopenia, *IABP* intra-aortic balloon pump, *IQR* interquartile range, *LAD* left anterior descending, *LVAD* left ventricular assist device, *MR* mitral regurgitation, *NR* not reported, *NSTEMI* non-ST elevation myocardial infarction, *OHT* orthotopic heart transplant, *POD* postoperative day, *pts* patients, *VAD* ventricular assist device

Despite the growing use of Impella in percutaneous coronary intervention with MCS, significant inconsistency exists in its application and outcomes, with increased adverse events, such as bleeding or thrombosis [4]. A survey revealed substantial differences in anticoagulation practices, with 41% of centers not adjusting heparin doses in purge solutions, potentially leading to complications [5]. Additionally, patients intolerant to or contraindicated against heparin faced challenges with conventional UFH solutions.

Beavers et al. [6] reported a retrospective study of 316 patients using BBPS as an alternative to heparin, demonstrating stability in purge pressures and flow rates, with no adverse events or deaths. These findings support the reliability of BBPS in managing bleeding risk and simplifying anticoagulation [7]. FDA approval for BBPS as an alternative to heparin in patients intolerant or contraindicated was received in April 2022 [8]. The efficacy of sodium bicarbonate in preventing catheter-related thrombosis in hemodialysis catheters further supports the use of BBPS [9]. The proposed mechanism of BBPS involves chelating calcium, neutralizing acidic pH, inhibiting fibrin aggregation, and improving blood protein stability without observable adverse effects [10, 11]. A case report in another country has also highlighted the success of BBPS in controlling bleeding and improving hemodynamics in patients with cardiogenic shock [12].

Impella users traditionally rely on systemic anticoagulation, mostly UFH, with a heparin concentration of 25–50 U/mL in the dextrose purge system. The Japanese on-label use involves adjusting the heparin concentration based on the patient’s condition [6, 13]. In our case, the off-label use of BBPS in Japan required urgent Ethics Committee approval at our institute. The switch to BBPS from UFH resolved Impella-related issues, allowing support for 52 days. In addition, concerns about purge system malfunction post-Impella 5.0 replacement align with our previous study [14], emphasizing the need for a standardized treatment strategy based on Impella 5.0 usage duration. Despite the challenges, transitioning from Impella CP to Impella 5.0 using BBPS maintained stability until the patient’s demise.

Furthermore, the difficulty in diagnosing HIT during Impella use due to fluctuating platelet counts and imbalances in the coagulation-fibrinolytic system [15], as well as the lack of experience with BBPS at our institution, may have contributed to the delay in the timing of initiation of BBPS use in this case. However, when compared with extracorporeal left VADs, the benefit of continued MCS with Impella using the BBPS appeared to outweigh the risk of thrombosis associated with HIT and bleeding associated with alternative MCS devices [16].

In conclusion, BBPS may be a superior alternative to conventional heparin for patients facing difficulties with heparin-purging fluids, especially those with HIT complications.

## Data Availability

The data underlying this article cannot be shared publicly for the privacy of the patient. The data will be shared on reasonable request to the corresponding author.
